# Effects of presurgical HIIT in hormone receptor‐positive, HER 2‐negative breast cancer cases: Two case reports from the GEICAM EFiK study

**DOI:** 10.14814/phy2.70511

**Published:** 2025-08-26

**Authors:** Txomin Pérez‐Bilbao, Sara López‐Tarruella, María Alonso‐Dueñas, Alejandro F. San Juan, Karla Ferreres‐García, Miguel Martín, Ana B. Peinado, Federico Rojo

**Affiliations:** ^1^ Health and Performance Department, Faculty of Physical Activity and Sports Sciences‐INEF Universidad Politécnica de Madrid Madrid Spain; ^2^ GEICAM Spanish Breast Cancer Group Madrid Spain; ^3^ Hospital General Universitario Gregorio Marañón Instituto de Investigación Sanitaria Gregorio Marañón (IiSGM), Universidad Complutense Madrid Spain; ^4^ Centro de Investigación Biomédica en Red de Oncología, CIBERONC‐ISCIII Madrid Spain; ^5^ Obstetrics and Gynecology Department Gregorio Marañón University General Hospital Madrid Spain; ^6^ LFE Research Group Universidad Politécnica de Madrid Madrid Spain; ^7^ Hospital Universitario Fundación Jiménez Díaz Madrid Spain

**Keywords:** breast neoplasms, case report, cell proliferation, exercise therapy, Ki‐67 antigen, preoperative exercise

## Abstract

The purpose of this study was to determine the effects of a high‐intensity interval‐training (HIIT) or stretching‐balance intervention 3 weeks prior to surgery in two hormone receptor‐positive and human‐epidermal growth‐factor receptor 2‐negative breast cancer patients. *Patient 1* (58 years, stage IIA ductal carcinoma) performed a preoperative exercise program, and *Patient 2* (63 years, stage IIB ductal carcinoma) underwent stretching‐balance intervention. Tumor Ki67, body composition, oxygen uptake, inflammatory biomarkers, and psychological variables were measured before the intervention (T1), and prior to tumor resection (T2). Ki67 remained unchanged in *Patient 2* and decreased by 28.6% in *Patient 1. Patient 1* maintained her body mass (BM) and body mass index (BMI) with minor variation, while *Patient 2* slightly increased her BM and BMI. Both patients increased their fat mass (FM) and %FM and decreased lean body mass (LBM) and %LBM, although to different extents. Peak oxygen uptake increased in *Patient 1 and decreased in Patient 2*. Interleukin‐6 decreased, and C‐reactive protein increased in both patients. *Patient 1* showed a reduction in depressive symptoms, while *Patient 2* exhibited no changes. Based on this case report, presurgical HIIT intervention appears to be a more promising exercise approach. However, larger scale‐controlled studies will be needed.

## Graphical abstract

Presurgical high‐intensity interval training (HIIT) may improve tumor proliferation (Ki67), body composition, VO^2^peak, IL‐6, and depression in breast cancer patients.

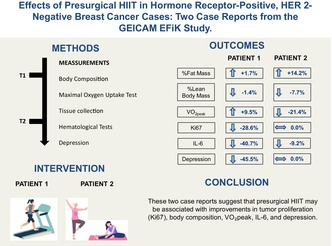

## 1 INTRODUCTION

Breast cancer (BC) was the most diagnosed cancer worldwide in 2022, accounting for 23.8% of all new cases in women and 11.5% in both sexes. It is also the leading cause of cancer death among women, accounting for 15.4% of all deaths (Ferlay et al., 2024).

Exercise is recognized as a safe (Singh et al., 2018) and effective tool for BC prevention (Guo et al., 2020) and for reducing recurrence and mortality (Chen, Irwin, et al., 2022). Several studies have reported impaired cardiorespiratory fitness (CRF), measured by V̇O_2max_ or V̇O_2peak_, in BC patients compared to healthy women (Jones et al., 2012; Peel et al., 2014). This is clinically relevant, as low CRF levels are linked to increased cancer‐related mortality among patients with cancer (Schmid & Leitzmann, 2015), including BC (Peel et al., 2009). High‐intensity interval exercise (HIIT) is a safe, feasible, and time‐efficient method to improve CRF in BC patients (Toohey et al., 2020; Tsuji et al., 2021). Notably, presurgical HIIT has been shown to improve CRF in cancer patients (Jones et al., 2007; Lavin‐Perez et al., 2021).

The biological processes underlying the benefits of exercise on cancer remain under study. Hypothesized mechanisms include reduced circulating inflammatory cytokines (e.g., interleukin‐6 [IL‐6], C‐reactive protein [CRP]) (Friedenreich et al., 2022) and decreased cancer cell proliferation (Dethlefsen et al., 2016; Friedenreich et al., 2022). Ki67 is a proliferation marker that provides information about tumor aggressiveness (Lukasiewicz et al., 2021; Nielsen et al., 2021; Parekh et al., 2018), treatment response, and recurrence risk (Lukasiewicz et al., 2021).

This study aimed to determine the effects of a preoperative exercise program on tumor proliferation (i.e., Ki67 protein level of expression) in hormone receptor‐positive (HR+)/human epidermal growth factor receptor 2‐negative (HER2−) early‐stage BC patients.

## 2 MATERIALS AND METHODS

### 2.1 Case description

This study was conducted in accordance with the Declaration of Helsinki, approved by the Ethics Committee from Hospital General Universitario Gregorio Marañón (ID: GEICAM/2014‐09), and registered at ClinicalTrials.gov (NCT03860740). Both patients provided written consent after being informed of all protocol‐related risks. The GEICAM/2014‐09 (EFiK) study was prematurely closed due to insufficient recruitment.

### 2.2 Patients

*Patient 1* was a 58‐year‐old woman with stage IIA ductal carcinoma of the right breast, HR+, HER2−, 35% Ki67 index, without axillary lymph node involvement. *Patient 2* was a 63‐year‐old woman with stage IIB ductal carcinoma of the left breast, stage IIB, HR+, HER2−, 30% Ki67 index, also without lymph node involvement.

### 2.3 Therapy and treatments

Both patients underwent lumpectomy and axillary lymph node dissection (ALND) followed by standard chemotherapy and at least 5 years of adjuvant endocrine therapy (ET).

Adverse events (AEs) were classified using the National Cancer Institute Common Terminology Criteria for AEs (NCI CTCAE) version 4.03.

*Patient 1* received fully planned chemotherapy and ET without dose modifications, while *Patient 2* required dose reductions due to related AEs. Specifically, *Patient 2* experienced grade 1 arthralgia; grade 2 nausea, vomiting, diarrhea, fatigue, and palmar‐plantar erythrodysesthesia syndrome (two different events); grade 3 respiratory infection; and grade 4 neutropenia. Treatment adjustments included dose reductions (for nausea, vomiting, diarrhea, respiratory infection, and second palmar‐plantar erythrodysesthesia syndrome), no dose change (for arthralgia, fatigue, and neutropenia), and treatment interruption (first palmar‐plantar erythrodysesthesia syndrome).

### 2.4 Exercise program interventions

Patients completed at least 10 of the 15 planned sessions within the 2–3 weeks prior to surgery.

*Patient 1* underwent 12 supervised medium‐high intensity treadmill sessions across 3 weeks. Five sessions were continuous medium‐high intensity of 25–35 min at 60%–75% of the heart rate reserve (HRR), and seven were interval‐based (25–35 min) with 30–40 s intervals at an intensity of 85%–100% of the HRR, interspersed with 1–2 min of active recovery.

*Patient 2* completed 10 sessions consisting of full‐body light static stretches (30 s per stretch), performed before the onset of discomfort and balance exercises (eyes open/closed, monopodal, 30 s each).

All sessions were supervised by a certified oncology exercise specialist, programmed according to each participant's cardiorespiratory level (measured by Peak Oxygen Uptake Test), and monitored using a Polar® S625XTM heart rate monitor (Polar Electro OY, Kempele, Finland).

### 2.5 Timeline

The study started 3 weeks before breast surgery. Patients were measured twice: T1 (baseline, before exercise/stretching) and T2 (after final session, before surgery). Tumor biopsies for Ki67 analysis were collected before the start of T1 and T2. Serum samples for inflammatory cytokines analysis were also collected at T1 and T2.

## 3 MEASUREMENTS AND ASSESSMENTS

### 3.1 Anthropometry and body composition

A Tanita BC 418 body composition analyzer (Tanita Corp., Tokyo, Japan) was used for bioelectrical impedance analysis (BIA). Measurements were taken at least 3 hours postprandial, with no vigorous exercise within the prior 12 h. Patients emptied their bladders before measurements. Body mass (BM), fat mass percentage (%FM), and lean body mass percentage (%LBM) were obtained. Height was measured using a SECA stadiometer (range 80–200 cm, Valencia, Spain). BMI was calculated as: body mass/height squared (kg/m^2^).

### 3.2 Peak oxygen uptake test

Peak oxygen uptake (V̇O_2peak_) was measured with a modified Bruce protocol to exhaustion on a computerized treadmill (H/P/COSMOS 3PW 4.0, H/P/Cosmos Sports & Medical, Nussdorf Traunstein, Germany). Expired gases were analyzed breath‐by‐breath (Jaeger Oxycon Pro, Erich Jaeger, Viasys Healthcare, Hochberg, Germany). Heart response was continuously monitored with a 12‐lead Jaeger electrocardiogram (Erich Jaeger, Hochberg, Germany).

### 3.3 Tissue collection and hematological tests

Ki67 expression was assessed by immunohistochemistry (IHC) in formalin‐fixed, paraffin embedded tumor tissue samples from T1 and T2 (Monoclonal Mouse Anti‐Human Ki‐67 Antigen, Clone MIB‐1, Dako Omnis‐Agilent) according to the manufacturer's recommendations and following the latest international recommendations for Ki67 assessment in BC (Nielsen et al., 2021). Ki67 IHC digital assessment was applied. IL‐6, ultra sensible C‐reactive protein (CRP) and TNFα were also analyzed in serum collected samples at T1 and T2. Additional Information about Ki67 and cytokine assessment is provided in Supplementary Materials.

### 3.4 Depression

The Center for Epidemiologic Studies‐Depression (CES‐D) Scale was developed to detect depressive symptomatology in the general population by measuring the frequency of events and ideas in the past week (Radloff, 1977). The CES‐D scale is a 20‐item instrument with each item rated on a 4‐point scale ranging from 0 (“rarely or none of the time”) to 3 (“most or all of the time”). The total score ranges from 0 to 60, and a higher score indicates a greater risk of depression. For the original CES‐D scale, a total score of 16 or higher is considered indicative of subthreshold depression (Radloff, 1977), and was the cut‐off used in this study. The CES‐D is a reliable and valid instrument in cancer patients (Park et al., 2023).

## 4 RESULTS

**FIGURE 1:**
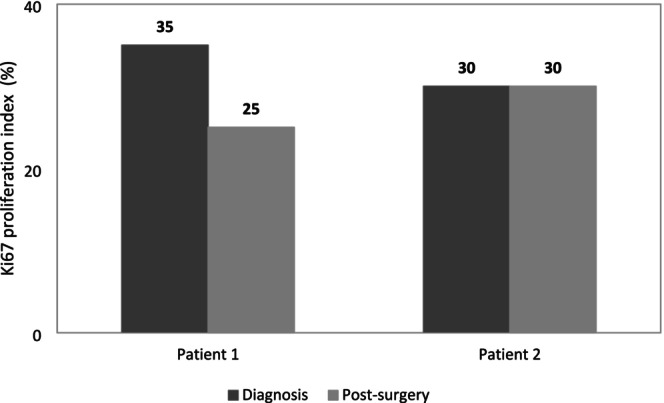
Ki67 protein (%) at diagnosis and post resection. Ki‐67 staining was assessed by evaluating 4–5 replicate fields per time point per patient, with approximately 500–1000 tumor cell nuclei counted per sample, in line with recommendations from the International Ki67 in Breast Cancer Working Group (Dowsett et al., 2011).

The results for anthropometric variables, body composition, V̇O_2peak_, Ki67, IL‐6, CRP protein, and depression from T1 to T2 are shown in (Tables 1 and 2, Figure 1).

**TABLE 1:** Results of anthropometry, body composition, V̇O_2peak_, and inflammatory cytokines before and after 3 weeks of intervention.

Variables	Patient case	T1	T2	%change from T1
BM (kg)	1	71,4	71,2	−0.3
	2	70,4	71,3	1.3
BMI (kg·m^−2^)	1	30,11	30,02	−0.3
	2	28,93	29,3	1.3
FM (kg)	1	28,8	29,3	1.7
	2	27,7	31,4	13.4
% FM	1	40,4	41,1	1.7
	2	39,3	44,9	14.2
LBM (kg)	1	42,6	41,9	−1.6
	2	42,7	39,9	−6.6
% LBM	1	59,66	58,85	−1.4
	2	60,65	55,96	−7.7
V̇O_2peak_ (mL·kg^−1^·min^−1^)	1	27,81	30,45	9.5
	2	27,74	21,8	−21.4
IL‐6 (pg/mL)	1	5,14	3,06	−40.7
	2	3,06	2,78	−9.2
TNFα (pg/mL)	1	8,29	6,85	−17.4
	2	7,15	5,52	−22.8
CRP (mg/L)	1	1,94	2,63	35.6
	2	1,47	1,76	19.7

Abbreviations: 1, patient 1, exercise intervention; 2, patient 2, control; BM, body mass; BMI, body mass Index; CRP, C‐reactive protein; FM, fat mass; IL‐6, interleukine‐6; LBM, lean body mass; T1, pre‐intervention; T2, post‐intervention; TNFα, tumor necrosis factor alpha; V̇O_2peak_, peak oxygen uptake.

**TABLE 2:** Results of depression.

Variables	Patient	T1	T2	%change from T1
CES‐D	1	11	6	−45.5
	2	1	1	0,0

Abbreviations: 1, patient 1, exercise intervention; 2, patient 2, control; CES‐D, Center for Epidemiologic Studies‐Depression Scale; T1, pre‐intervention; T2, post‐intervention.

*Patient 1* maintained BM and BMI with minimum change, while *Patient 2* showed slight increases. Both patients experienced increases in FM and %FM and decreases in LBM and %LBM. V̇O_2peak_ increased in *Patient 1* and declined in *Patient 2*.

Ki67 remained unchanged in *Patient 2* and decreased in *Patient 1*. IL‐6 levels decreased, and CRP levels rose in both patients.

*Patient 1* showed a reduction in depressive symptoms, while *Patient 2* exhibited no changes.

## 5 DISCUSSION

The purpose of our study was to assess the effects of a preoperative exercise program prior to surgery, as the first therapeutic intervention, on tumor proliferation (i.e., Ki67 protein expression) in recently diagnosed HR+/HER2− early‐stage BC patients.

After the intervention, BM and BMI minimally decreased in *Patient 1* (0.3% for both parameters) and increased in *Patient 2* (1.3% each). Both patients showed increased FM and %FM, and decreased LBM and %LBM, the increase in FM and %FM being eight times higher in *Patient 2* than in *Patient 1*, while the decrease in LBM and %LBM experienced by *Patient 1* was approximately five times lower than in *Patient 2*.

Previous studies observed a decrease in BM (0.2–0.4 kg) and FM (0.5–0.6 kg) respectively, alongside increases in LBM (0.1–0.4 kg) (Gillis et al., 2019; Schmitt et al., 2016). In the present case series, both patients lost LBM, although less pronounced in *Patient 1*, likely reflecting the muscle‐protective effect of exercise described in the literature (Gillis et al., 2019; Schmitt et al., 2016). Including strength training might have yielded better results, as it is known to counteract muscle loss in cancer patients (Koeppel et al., 2021).

Regarding V̇O_2peak_, *Patient 1* improved by 9.5%, whereas it decreased by 21.4% in *Patient 2*. These results were consistent with prior studies in cancer patients (Banerjee et al., 2018), (Dunne et al., 2016), (Karenovics et al., 2017). Possible mechanisms behind V̇O_2peak_ improvement include increased stroke volume, plasma volume, muscle oxidative capacity, and/or maximal arteriovenous oxygen difference (Astorino et al., 2017; Daussin et al., 2007; Jacobs et al., 2013; Matsuo et al., 2014).

Ki67 levels remained unchanged in *Patient 2*, while decreased by −28.6% in *Patient 1*. In the studies by Rao et al. (Rao et al., 2012) and Ligibel et al. (2019), women with BC underwent preoperative concurrent training, and both the control group and the exercise group experienced a decrease in Ki67 levels. While Ligibel et al. (2019) found no significant group differences, Rao et al. (2012) reported a great decrease of 79.4% in the exercise group, with a lower diminution in the control group (31.0%). Our results align with Rao's study, although at a lower magnitude.

While these findings are promising, a single Ki67 assessment by immunohistochemistry at each time point may present variability, especially across platforms and pathologists. To minimize this, several quality control strategies were implemented in the present study, including strict procedural standardization, centralized analysis in an ISO 15189‐accredited diagnostic laboratory, use of an automated IHC staining system, and inclusion of both internal and external quality controls. Additionally, all samples were evaluated by a pathologist with recognized expertise in breast cancer and Ki67 interpretation. Despite these precautions, the results must still be interpreted with appropriate caution due to the exploratory nature of the study.

In terms of inflammatory cytokines, IL‐6 decreased by 40.7% in *Patient 1*, and 9.2% in *Patient 2*. On the other hand, CRP increased in both patients, but unevenly (i.e., *Patient 1*, 35.6%, *Patient 2*, 19.7%). These results agree in the case of IL‐6, but not in CRP, with those found by Jones et al. (2009) in lung cancer patients, which accomplished a combination of HIIT and moderate intensity cardiorespiratory exercise 4–6 weeks before surgery. They observed that IL‐6 and CRP levels decreased 3.7% and 19%, respectively. The reason for this disagreement could be that exercise with a duration of <16 weeks has not been shown to reduce CRP levels in BC patients (Abbasi et al., 2022). On the other hand, our results for IL‐6 are in line with the conclusions of the systematic review conducted by Abbasi et al. (2022), which showed that short exercise interventions in BC patients produced a significant diminution of IL‐6 levels.

Several investigations indicate that HIIT exercise has an inhibitory effect on proliferation in different types of cancer, including BC in humans (Bettariga et al., 2024) and mice (Jee et al., 2022; Khoramipour et al., 2024). Moreover, HIIT improves the quality of life and decreases the adverse effects associated with treatment in patients with BC (Klavina et al., 2024). In the case of IL‐6, it is known that high levels are associated with poor prognosis and reduced survival in patients with BC (Chen, Wei, et al., 2022; Tawara et al., 2019). Finally, in relation to depression, *Patient 1* showed a significant decrease of 45.5% in CES‐D score, while *Patient 2* did not experience any changes in depression levels. These results of depression are consistent with Chen et al. (2023), where they found that HIIT interventions in BC patients could lower depression levels. A review by Haroon et al. (2012) may explain a possible mechanism underlying positive effects of exercise. They affirmed that inflammatory cytokine levels may induce the central nervous system to generate symptoms of depression. The anti‐inflammatory effect of exercise could play a beneficial effect over these symptoms.

In summary, the observed improvements in Ki67 levels, body composition, V̇O_2peak_, IL‐6, and depression in these two patients appear to align with trends reported in prior research (Banerjee et al., 2018; Chen, Wei, et al., 2022; Dunne et al., 2016; Karenovics et al., 2017; Khoramipour et al., 2024; Klavina et al., 2024; Rao et al., 2012; Tawara et al., 2019).

In conclusion, these two case reports describe the individual responses to a presurgical HIIT or stretching‐balance intervention in two HR+/HER2‐ early‐stage BC patients, on tumor proliferation, body composition, oxygen uptake, systemic inflammatory biomarkers, and psychological variables. These findings suggest that presurgical HIIT intervention may improve tumor proliferation (Ki67), body composition, oxygen uptake, inflammatory response (IL‐6), and depression levels. Larger prospective research is required to confirm these preliminary findings and evaluate the impact on patient prognosis.

## FUNDING INFORMATION

This work was supported by the FSEOM/Font‐Vella Grant from the Spanish Society of Medical Oncology in collaboration with the mineral water company Fontvella, and by the Spanish Breast Cancer Group (GEICAM) (Project code GEICAM/2014‐09).

## CONFLICTS OF INTEREST

No conflicts of interest, financial or otherwise, are declared by the authors.

## Supporting information


Data S1.

## Data Availability

The data that support the findings of this study are available from the corresponding author upon reasonable request.
