# Supplementary material for: Effects of presurgical HIIT in hormone receptor‐positive, HER 2‐negative breast cancer cases: Two case reports from the GEICAM EFiK study
Source: Physiol Rep. 2025 Aug 26;13(16):e70511. doi: 10.14814/phy2.70511 (PMC12380710; doi:10.14814/phy2.70511)
Supplement: Supplementary file 1 — Data S1. [file PHY2-13-e70511-s001.docx]

**Additional information about Ki67 and cytokine assessment.**

For the Ki-67 IHC assay, Ki67 nuclear protein expression was assessed in formalin-fixed, paraffin-embedded tumor tissue samples obtained at T1 and T2 timepoints. The analysis was performed by immunohistochemistry according to the manufacturer's recommendations and in line with the most recent international guidelines for Ki67 assessment in breast cancer (Nielsen TO et al., Journal of the National Cancer Institute, 2021). The staining procedure was conducted on the Dako Omnis (Agilent Technologies, Carpinteria, CA) platform using a validated, automated staining protocol. Heat-induced epitope retrieval was performed using EnVision FLEX Target Retrieval Solution, Low pH (GV805, Dako Onmis). After deparaffinization and rehydration, specimens were incubated with a monoclonal mouse anti-human Ki-67 antibody (Monoclonal Mouse Anti-Human Ki-67 Antigen, Clone MIB-1, Dako Omnis-Agilent), followed by a visualization reagent containing a secondary antibody conjugated with horseradish peroxidase (EnVision FLEX Visualization Reagent). The chromogenic substrate 3,3′-diaminobenzidine (DAB) was then applied, and sections were counterstained with hematoxylin and coverslipped. A high-sensitivity EnVision FLEX detection system was used (Dako Omnis). Table 1 summarizes the equipment and reagents employed in the Ki67 immunohistochemistry assay, including their identification details (e.g., catalogue, lot, and serial numbers) for precise traceability.

| **Table 1. Identification details of equipment and reagents used for Ki67 immunohistochemistry.** | | |
| --- | --- | --- |
| **Instrument** | **Code No.** | **Serial No.** |
| Dako Omnis | NA | 170003966 |
| **Reagent** | **Code No.** | **Lot No.** |
| Envision™ FLEX Wash Buffer, 20X | GC807 | 41541785 |
| EnVisionTM FLEX Peroxidase Blocking Reagent | DM841 (GV800) | 41522558 |
| EnVisionTM FLEX/HRP | DM842 (GV800) | 41525903 |
| EnVisionTM FLEX DAB+ Chromogen | DM847 (GV800) | 41529095 |
| EnVisionTM FLEX Substrate Buffer | DM843 (GV800) | 41522512 |
| Ki-67 Antigen, Clone MIB-1, FLEX RTU | GA626 | 41523497 |
| Envision™ FLEX TRS, Low pH 50X | GV805 | 84949444 |
| Dako Hematoxylin | GC808 | 41525911 |

Cytokines were analyzed using chemiluminescence. As stated earlier, duplicate analysis is not necessary for this technique. The equipment used (Immulite 2000 and Immulite One from Siemens) is highly precise, and daily analytical controls are performed to ensure consistency and accuracy of results.

Reagent kit reference used for PCR and TNFα determination, corresponding to samples from patients EFIK1 and EFIK2, analyzed in April 2023 as part of the GEICAM/2014-09 (EFIK) study, were:

- PCR: Atellica CH High Sensitivity C-Reactive Protein (hsCRP). Reference: 11097633
- TNFα: Immulite. Reference: 6602826 LKNF1
